# Chitosan-encapsulated *Aloe vera* nanoparticles outperform carrier-free forms in enhancing MSCs therapy for amikacin nephrotoxicity

**DOI:** 10.1038/s41598-025-20918-6

**Published:** 2025-10-01

**Authors:** Aya Tawfik, Alyaa Farid

**Affiliations:** Faculty of Science, Cairo University, Giza, Egypt

**Keywords:** Amikacin, Chitosan, *Aloe vera*, Nanoparticles, MSCs, Biotechnology, Drug discovery, Materials science, Nanoscience and technology

## Abstract

Acute kidney injury (AKI) induced by nephrotoxic drugs like amikacin remains a clinical challenge, with mesenchymal stem cells (MSCs) showing limited efficacy due to poor survival and engraftment. While *Aloe vera* extract (AVE) possesses renoprotective properties, its bioactive compounds suffer from low bioavailability and instability. The study developed and characterized two nanoformulations of AVE; carrier-free nanoparticles (AVENPS) and chitosan-encapsulated nanoparticles (AVE-CSNPS), to enhance mesenchymal stem cells (MSCs) therapy for amikacin-induced kidney injury. In vitro bioactivities (antioxidant, anti-inflammatory, anticoagulant, and cytotoxicity) were evaluated for both nanoformulations. For in vivo assessment, amikacin-induced AKI mice received: MSCs alone, MSCs + AVE, MSCs + AVENPS, MSCs + CSNPS or MSCs + AVE-CSNPS. Phytochemical characterization revealed both formulations preserved key bioactive compounds, with AVE-CSNPS showing superior retention of flavonoids and essential minerals. Physicochemical analysis demonstrated AVE-CSNPS had optimal characteristics for drug delivery, including larger hydrodynamic size, higher positive zeta potential, and enhanced stability (PDI < 0.3). In vitro, AVE-CSNPS exhibited significantly stronger antioxidant, anti-inflammatory, and anticoagulant effects compared to AVENPS, while showing lower cytotoxicity. *In vivo*, the MSCs + AVE-CSNPS combination therapy most effectively restored kidney function, normalized oxidative stress markers and pro-inflammatory cytokines. These results demonstrated chitosan encapsulation significantly enhanced *Aloe vera*’s therapeutic potential and MSCs synergy, offering a promising nano-enabled strategy for renal regeneration.

**Supplementary Information:**

The online version contains supplementary material available at 10.1038/s41598-025-20918-6.

## Introduction

 Acute kidney injury (AKI) arises from diverse etiologies, including ischemia, drug-induced nephrotoxicity, diabetes, hypertension, autoimmune disorders, and genetic defects^1^. Exposure to nephrotoxic agents, such as heavy metals, mycotoxins, and medications like nonsteroidal anti-inflammatory drugs (NSAIDs), further exacerbate renal damage^2^. Aminoglycosides (AGs), a class of antibiotics introduced in the 1940 s, remain critical for treating severe gram-negative bacterial infections, particularly multidrug-resistant strains^3,4^. Their bactericidal action involves binding to the 30 S ribosomal subunit, disrupting bacterial protein synthesis^5^. Despite their efficacy, AGs like gentamicin, amikacin, and tobramycin lead to significant nephrotoxic risks, with AKI incidence ranging from 5 to 25% after 5–7 days of treatment^6–11^. AGs accumulate in proximal tubular cells, inducing vasoconstriction and tubular necrosis, underscoring the need for strategies to mitigate renal toxicity^7,8^.

Current AKI treatments rely on antioxidants, angiotensin receptor blockers (ARBs), and angiotensin-converting enzyme inhibitors (ACEIs), which modulate blood pressure and oxidative stress^12^. However, these therapies face limitations, including variable patient responses, side effects, and incomplete resolution of renal pathology^13,14^. Left untreated, AKI progresses to chronic kidney disease (CKD) and end-stage renal failure, necessitating kidney transplantation, a gold standard for restoring function and improving quality of life^15,16^. Yet, donor scarcity, immune rejection, and reliance on immunosuppressants hinder transplantation success^17,18^, prompting the exploration of alternative therapies.

Mesenchymal stem cells (MSCs) offer promise due to their immunomodulatory and regenerative properties, including differentiation into renal cell lineages and secretion of reparative factors^19,20^. Preclinical studies demonstrate MSCs efficacy in AKI models, and early clinical trials are underway^21^. However, challenges persist, such as senescence, where inflammatory damage and oxidative stress impair MSCs function, accelerating aging and reducing therapeutic potential^22–24^; and safety concerns as risks include uncontrolled proliferation, tumorigenicity, and inconsistent *in vivo* engraftment^25,26^. To address these limitations, combining MSCs with bioactive components may enhance renoprotection by improving MSCs viability, targeting oxidative stress, and amplifying anti-inflammatory effects.

Herbal remedies have been integral to traditional medicine for centuries, leveraging synergistic bioactive compounds to promote healing^27^. Their complex phytochemical profiles enhance conventional treatments, prompting modern research to explore their therapeutic potential. Notably, the National Institutes of Health reports that 27 of 680 clinical trials on MSCs incorporate herbal supplements, highlighting their growing relevance^28^. Specific compounds, such as blueberry extract, matcha powder, carnosine, catechins, and vitamin D, have been shown to stimulate stem cell proliferation^29^. For instance, oleic and linoleic acids enhance hematopoietic stem cell development^30^, while plant extracts improve MSCs differentiation, proliferation, and anticancer efficacy in vitro^31^.

Among medicinal plants, *Aloe vera* (*Aloe barbadensis* Miller) stands out for its multifaceted applications. This succulent plant, native to arid regions worldwide, features fleshy leaves composed of three layers: an outer peel that synthesizes amino acids and sugars, an inner gel that is rich in water, glucomannan, fatty acids, phytosterols, and vitamins, and central latex that contains anthraquinones and flavonoids^32^. Historically, *Aloe vera* has been used for wound healing and dermatological conditions across ancient civilizations, including Egypt, Greece, and China^33,34^. Its modern recognition began in the 1930 s for treating radiation dermatitis^35^, underscoring its enduring therapeutic value.

Nanoparticles offer a transformative platform for enhancing herbal drug delivery. They enable targeted administration and sustained release of bioactive compounds, minimizing side effects while maximizing efficacy^35,36^. Plant extracts can be directly converted into nanoparticles (NPs) without a carrier through precipitation, solvent evaporation, or supercritical fluid technology^37^. These techniques rely on reducing the particle size of bioactive compounds via physical or chemical processes, such as antisolvent addition or pH-driven self-assembly^38^. For example, hydrochloric acid-induced precipitation can synthesize plant extract nanoparticles, where bioactive compounds aggregate into nanoscale particles^39^. However, carrier-free NPs often exhibit lower stability, uncontrolled burst release, and reduced bioavailability due to the lack of a protective matrix^40^. They may also suffer from rapid physiological degradation, limiting their therapeutic efficacy. When comparing carrier-free NPs with carrier-based NPs, key differences emerge in encapsulation efficiency, stability, and bioactivity. Carrier-free NPs typically show moderate encapsulation efficiency and faster release kinetics, whereas carrier-based NPs achieve higher encapsulation and sustained release due to the polymer matrix^41^.

Chitosan, a biodegradable and biocompatible polysaccharide derived from chitin, is an exceptional carrier for plant extracts due to its mucoadhesive properties, positive charge, and ability to enhance drug stability and bioavailability^42^. Its amine groups enable electrostatic interactions with bioactive compounds, improving encapsulation efficiency and controlled release^43^. Studies demonstrate that chitosan nanoparticles protect phytochemicals from degradation, enhance cellular uptake, and reduce toxicity, making them ideal for delivering plant-based therapeutics^44^. For instance, chitosan-encapsulated curcumin showed improved anti-inflammatory effects compared to free curcumin^45^. Additionally, chitosan’s inherent antimicrobial, antioxidant, and anti-inflammatory properties synergize with plant extracts, further enhancing therapeutic outcomes^46,47^.

This study aimed to develop an innovative therapeutic approach for amikacin-induced AKI by combining the regenerative potential of MSCs with the enhanced delivery of *Aloe vera* bioactive compounds through nanotechnology. The study fabricated and characterized two NPs formulations: 1- carrier-free *Aloe vera* nanoparticles (AVENPS) prepared via acid precipitation method and 2- *Aloe vera* extract loaded-chitosan nanoparticles (AVE-CSNPS) using ionic gelation method. The two NPS formulations were compared by assessing encapsulation efficiency, stability, antioxidant, anti-inflammatory, and synergistic effects with MSCs on renal function recovery *in vivo*. By investigating this combinatorial therapy, the study addresses critical limitations in current AKI treatment, including the short lifespan and reduced functionality of MSCs in inflammatory microenvironments, while enhancing the bioavailability and targeted delivery of *Aloe vera’s* bioactive components. This integrated investigation bridges the fields of herbal medicine, nanomedicine, and regenerative therapy, with potential implications for improving outcomes in drug-induced nephrotoxicity and other forms of AKI.

## Materials and methods

### Aloe vera extraction method

*Aloe vera* (*Aloe barbadensis*) leaves were collected from the garden of the Faculty of Agriculture, Cairo University. The plant has a height of 50 cm and was 2 years old and was identified by an expert botanist. A voucher specimen was placed in the herbarium of the Department of Botany, Faculty of Science, Cairo University, Egypt. The plant name was checked with http://www.theplantlist.org. Briefly, the dried leaves were ground into a fine powder, followed by homogenization and maceration of 39 g of leaves powder with 400 ml of 85% ethanol in a sealed container, then left for 7 days at room temperature. The mixture was filtered under vacuum, yielding 11 g of *Aloe vera* extract (AVE).

### Nanoparticles Preparation

#### Aloe vera extract nanoparticles (AVENPS)

Five g of AVE powder were weighed and placed in a container with 20 mL of 38% HCl solution^39^. This mixture was stirred on a magnetic stirrer while keeping the temperature at 30 ± 2 °C until the formation of nanoparticles. The resulting AVENPS suspension was subjected to purification via dialysis against deionized water using a cellulose membrane (12–14 kDa) for 24 h to remove free acid, HCl salts, and any small molecular weight impurities. The dialyzed suspension was then lyophilized to obtain the solid AVENPS powder. For all biological experiments (in vitro assays and *in vivo* administration), this lyophilized AVENPS powder was re-dispersed in the appropriate physiological buffer. The pH of the final dispersion was confirmed to be within the physiological range (7.0-7.4) using a calibrated pH meter immediately before use.

While numerous advanced methods for generating carrier-free nanoparticles exist- such as flash nanoprecipitation for superior size control^48^, supercritical antisolvent (SAS) precipitation for high purity and scalability^49^, nanoemulsion templating^50^, and high-pressure homogenization^51^- the acid precipitation method was specifically chosen for this study. This technique is a well-documented and straightforward approach for inducing the self-assembly of phytochemicals into nanoscale particles through a pH-shift mechanism^39,52^. Its selection was strategic for three primary reasons: First, it enabled a direct and unambiguous comparison by providing a pristine benchmark of what nano-sizing alone can achieve, allowing us to isolate and quantitatively demonstrate the added value of chitosan encapsulation. Second, its cost-effectiveness, minimal equipment requirements, and high reproducibility^39,52^ made it an ideal practical benchmark against which to compare the more complex ionic gelation process. Finally, as a recognized technique in nanophytopharmacology, it allows our work to build effectively upon prior comparative formulation research^39,52^.

#### Aloe vera extract loaded Chitosan nanoparticles (AVE-CSNPS)

AVE-CSNPS were prepared using a modified ionic gelation method optimized for bioactive compounds encapsulation^47,53,54^. The ionic gelation method was selected due to its numerous advantages for bioactive encapsulation. These include its mild, aqueous processing conditions which are essential for preserving the stability of thermolabile phytochemicals in the *Aloe vera* extract, its high encapsulation efficiency driven by electrostatic interactions between chitosan and anionic bioactives, and its overall simplicity, reproducibility, and scalability using biocompatible materials. First, medium molecular-weight chitosan (75% deacetylated, Sigma-Aldrich) was dissolved in 1% (v/v) acetic acid solution (pH 5.0) at 0.5% (w/v) concentration under magnetic stirring (800 rpm, room temperature, 2 h) until complete dissolution. For active loading, 10 mg of AVE was dissolved in 10 mL of chitosan solution and stirred for 1 h at room temperature (25 ± 2 °C) to ensure molecular interaction. The crosslinking process was initiated by dropwise addition of 0.1% (w/v) sodium tripolyphosphate (TPP) solution (chitosan: TPP ratio 5:1) at a rate of 1 mL/min using a peristaltic pump under constant sonication (20 kHz, 50% amplitude, 5 min pulse cycles). The resulting suspension was stirred for an additional 30 min to complete nanoparticle formation, followed by pH adjustment to 5.5 with 0.1 M NaOH. The nanoparticles were purified by ultracentrifugation (15,000 rpm, 4 °C, 30 min), washed 3 times with deionized water to remove unencapsulated AVE, lyophilized and stored. The hydrodynamic size and zeta potential of nanoparticles were determined.

### Determination of total phenolic content (TPC)

The TPC of AVE, AVENPS and AVE-CSNPS was determined using the Folin-Ciocalteu colorimetric method. Samples were prepared by dissolving 1 mg/mL in methanol, followed by centrifugation (10,000 × g, 10 min) and filtration to remove particulates. The assay was performed by mixing 100 µL of sample with 500 µL of freshly prepared 10% Folin-Ciocalteu reagent, vortexing for 30 s, and incubating for 5 min at 25 °C in the dark. Subsequently, 400 µL of 7.5% sodium carbonate solution was added, and the mixture was incubated for 60 min. The result was read at 765 nm and expressed as mg GAE/g.

### Determination of total flavonoid content (TFC)

The TFC of the samples (AVE, AVENPS and AVE-CSNPS) was quantitatively determined using the aluminum chloride colorimetric method with quercetin as the reference standard. In brief, 0.5 mL of diluted sample was mixed with 1.5 mL of 95% ethanol, followed by addition of 0.1 mL of 10% aluminum chloride solution, 0.1 mL of 1 M potassium acetate, and 2.8 mL of distilled water. The reaction mixture was vortexed thoroughly and allowed to stand at room temperature for 30 min in the dark to allow for complete color development. The absorbance was then measured at 415 nm and the result was expressed as mg QE/g.

### Determination of encapsulation efficiency (EE%) and drug loading (DL%)

The EE% and DL% capacity of AVE-CSNPS was evaluated using validated analytical methods^54^. For EE% determination, the unencapsulated AVE was separated from the nanoparticle suspensions through ultracentrifugation (20,000 × g, 45 min at 4 °C) using Amicon Ultra centrifugal filters (100 kDa MWCO). The filtrate containing free AVE compounds was analyzed for TPC using the Folin-Ciocalteu method and for TFC via aluminum chloride colorimetry, with results compared against the initial AVE solution. The encapsulation efficiency was calculated as the following: EE% (TPC) = (TPC in nanoparticles/TPC in initial extract) × 100 or EE% (TFC) = (TFC in nanoparticles/TFC in initial extract) × 100. For drug loading determination, the nanoparticle pellets were washed three times with deionized water, lyophilized, and precisely weighed. DL% was calculated using: DL% = (Mass of encapsulated AVE/Total mass of nanoparticles) × 100. The mass of encapsulated AVE was determined through spectrophotometric method [complete nanoparticle dissolution in 0.1 M NaOH followed by absorbance measurement at 220 nm^54^. For AVENPS, retention efficiency was calculated to quantify the recovery of bioactive compounds in nanoparticulate form, as classical EE% and DL% metrics are not applicable. The yield of AVENPS was calculated: Yield % = (Mass of lyophilized AVENPS/Mass of initial AVE) x 100.

For the AVE-CSNPS, classical encapsulation efficiency (EE%) and drug loading (DL%) were determined, as a distinct polymer carrier matrix entraps the bioactive compounds. In contrast, the carrier-free AVENPS are composed solely of self-assembled *Aloe vera* bioactives without an external matrix, making the concepts of EE% and DL% mathematically inapplicable. Therefore, for AVENPS, the retention efficiency was calculated to quantify the recovery of bioactive compounds in nanoparticulate form after the precipitation process, serving as the appropriate analogous metric.

### Characterization of AVE and AVENPS

#### Gas chromatography–mass spectrometry (GC–MS) analysis

The phytochemical profiles of AVE, AVENPS and AVE-CSNPS were characterized using a Thermo Scientific Trace GC1310-ISQ mass spectrometer equipped with an advanced TG-5MS fused silica capillary column (30 m × 0.25 mm ID, 0.25 μm film thickness). The analytical method was optimized for maximum resolution of bioactive compounds with the following temperature program: initial oven temperature: 50 °C (held for 2 min), ramp 1: 10 °C/min to 230 °C (held for 2 min) and ramp 2: 5 °C/min to 290 °C (held for 2 min final elution). Ultra-high purity helium carrier gas was maintained at a constant flow rate (1.0 mL/min) to ensure optimal peak separation. The injector port was set at 250 °C in split mode (20:1) with a 1 µL injection volume, while the mass spectrometer interface and ion source were stabilized at 260 °C to prevent condensation of high-boiling-point compounds. To minimize solvent interference, a 3-minute solvent delay was used prior to mass spectral acquisition. The ISQ single quadrupole mass detector operated in electron impact mode at 70 eV, scanning a mass range of 50–650 m/z with a 0.2 s/scan rate. System calibration was performed using perfluorotributylamine (PFTBA) for mass accuracy verification. Data acquisition and processing were conducted via Thermo Scientific Xcalibur™ software (v4.0), with compound identification achieved by matching mass spectra against the NIST 17 Mass Spectral Library (match threshold > 85%) and verified with authentic standards when available.

#### High performance liquid chromatography (HPLC) analysis

The phenolic and bioactive component profiles of AVE, AVENPS and AVE-CSNPS were analyzed using a HPLC system (Agilent 1260 series) equipped with a quaternary pump, thermostatted column compartment, and diode array detector (DAD). Separation was achieved on an Eclipse Plus C18 reversed-phase column (4.6 × 100 mm, 3.5 μm particle size) maintained at 40 °C for optimal peak resolution and reproducibility. The mobile phase consisted of (A) 0.1% trifluoroacetic acid (TFA) in ultrapure water and (B) acetonitrile, delivered at a constant flow rate (0.9 mL/min) using the following linear gradient elution program: 0–5 min: 82% A → 80% A, 5–8 min: 80% A → 60% A, 8–12 min: 60% A → 82% A and 12–20 min: 82% A (column re-equilibration). A 5 µL volume of filtered sample was injected in partial loop mode to ensure precision. Detection was performed at 280 nm using DAD, with simultaneous monitoring at 254 and 320 nm to confirm peak purity. Data acquisition and processing were conducted using Agilent OpenLAB CDS ChemStation software, with peak identification based on retention time matching and UV spectral comparison against authenticated reference compounds.

#### Inductively coupled plasma (ICP) analysis

For determination of the elemental composition in AVE, AVENPS and AVE-CSNPS, ICP was used after an ashing process. Dried samples (2.5 g) was burned in stages up to 570 °C, then the produced ash was treated with a mixture of acids (HCl and HNO_3_) and H_2_O_2_, dried, and dissolved in 1 M HNO_3_. After centrifugation, the element concentrations were measured using ICP.

### In vitro characterization

For both the coagulation and anti-inflammatory assays, fresh rat blood was collected in-house from healthy adult male Sprague Dawley rats (weighing 200–250 g) bred in the institutional animal facility of Faculty of Science, Cairo University. The rats were housed under standard conditions (12-h light/dark cycle, 25 ± 2 °C, 50–60% humidity) with ad libitum access to water and chow. Blood was drawn via cardiac puncture under anesthesia (50 mg/kg sodium pentobarbital) following the approved protocol (CU-IACUC approval no. CUIF6122). Heparinized blood (for anti-inflammatory assays) and citrate-anticoagulated plasma (for coagulation tests) were prepared immediately after collection. All procedures adhered to the ARRIVE guidelines and institutional ethical standards for animal welfare, minimizing discomfort and sample variability. Rats were used instead of mice for practical considerations where larger blood volume/animal (rats vs. mice) allowed for sufficient sample replicates without requiring excessive animal numbers. All in vitro assays were validated to ensure reproducibility and sensitivity. Reproducibility was confirmed by performing each experiment in triplicate (*n* = 3) with results presented as mean ± standard deviation (SD). Sensitivity was established by employing a broad range of sample concentrations to generate comprehensive dose-response curves, allowing for the detection of precise efficacy differences between formulations. Standard reference controls were included in each assay run to validate system performance.

#### In vitro antioxidant activity

The DPPH antioxidant assay is a gold-standard, widely validated method in phytochemistry. Our protocol followed the classic methodology of Blois^55^ with precise incubation conditions. The DPPH (2,2-Diphenyl-1-picrylhydrazyl) free radical scavenging experiment was employed to determine the antioxidant activities of AVE, AVENPS, and AVE-CSNPS. The protocol began by combining 1 ml of 0.1 mM DPPH in ethanol with different concentrations of samples (from 1.95 to 1000 µg/ml). The reaction mixtures were vortexed (30 s) and incubated in darkness at 25 ± 0.5 °C for precisely 30 min in a temperature-controlled environment. The absorbance was measured at 517 nm to calculate the DPPH scavenging activity, comparing the sample and control absorbance (Scavenging % = (absorbance of control - absorbance of sample)/absorbance of control × 100).

#### In vitro coagulation activity

The clotting assays [PT (prothrombin time) and PTT (partial thromboplastin time)] were conducted using commercial diagnostic kits from recognized manufacturers, which are pre-validated for accuracy and reproducibility in clinical and research settings. The use of citrate-anticoagulated rat plasma and strict temperature control (37 °C) are critical standard practices for these assays^56^. This dual-assay approach is critical as it probes both the extrinsic (PT) and intrinsic (PTT) coagulation pathways, providing a complete preliminary safety profile and mechanistic insight into any observed anticoagulant effects prior to in vivo administration.The anticoagulant activities of AVE, AVENPS, and AVE-CSNPS were assessed by measuring the clotting time with heparin as the standard. The manufacturer’s instructions for using PT and PTT reagents were followed. 900 µL of rat plasma was mixed with 100 µl of heparin or varying concentrations of the samples (25 to 75 µg/mL) at 37 °C and then the clotting time was recorded in seconds.

#### In vitro anti-inflammatory (membrane stabilization) assay

The hemolysis (membrane stabilization) assay is a recognized model for estimating anti-inflammatory activity in vitro^57^. The use of fresh rat erythrocytes and careful control of hypotonicity ensured a sensitive and reproducible system. Three ml of fresh rat’s blood (collected on heparin) was used for the preparation of red blood cells suspension by centrifugation at 1500 rpm for half an hour. The resulting pellet was suspended in phosphate buffered saline (PBS). Various concentrations of AVE, AVENPS, and AVE-CSNPS (ranging from 100 to 1000 µg/mLl) were prepared followed by dilution, individually, in 5 mL of distilled H_2_O or PBS (hypotonic and isotonic solutions, respectively). Indomethacin at 200 µg/mL was used as the standard. Red blood cells suspension (0.1 mL) was mixed with the samples, followed by incubation for 60 min at 37 °C and centrifugation for 10 min at 1500 rpm. Hemoglobin release in the supernatant was measured at 540 nm. The percentage of hemolysis inhibition was calculated using the formula: 1 - [(absorbance in hypotonic solution – absorbance in isotonic solution)/(absorbance of control - absorbance in isotonic solution)] × 100.

#### In vitro cytotoxicity (MTT) assay

HEK-293 cell line (100 µL/well, 10^5^ cells) were cultured for twenty-four hours at 37 °C to form the monolayers. Various concentrations of AVE, AVENPS, and AVE-CSNPS (31.25 to 1000 µg/ml) were mixed with RPMI medium and added to the cultured cells followed by another twenty-four hours incubation. Twenty µL of MTT (3-(4,5-dimethylthiazol-2-yl)−2-5-diphenyltetrazolium bromide) at a concentration of 5 mg/mL was added to the cultured cells followed by four hours of incubation at 37 °C in 5% CO_2_. Dimethylsulfoxide (DMSO) was used to dissolve the resulted formazan, and the result was recorded at an absorbance of 560 nm.

### Bone marrow-mesenchymal stem cells

The mouse BM-MSCs cells (sixth passage) were purchased from Cyagen, USA (MUBMX-01001). BM-MSCs were isolated from the C57BL/6 mouse’s healthy bone marrow, demonstrating a strong differentiation and proliferation capacity. Following the manufacturer’s guidelines, the flow cytometry findings indicated that CD117 and CD31 were negative (< 5%). Conversely, more than 70% of Sca-1, CD29, and CD44 tests were positive.

### Experimental design

Male BALB/c mice were purchased from the animal house colony, National Research Centre, Cairo, Egypt; and kept under standard experimental condition (22 ± 2 °C, 12 h dark/light cycle and 50 ± 2% humidity) for one week for acclimatization. Animals were fed standard mouse chow and water ad libitum, throughout the experimental time; and the work was performed in line with the ARRIVE guidelines 2.0. The sample size was calculated by power analysis method and approved by the institutional animal care and use committee of Cairo University (CU-IACUC) under the number (CUIF6122). The required number of animals in each group have to be < 6 to accomplish 80% power with a 0.05 significance. The researchers performing the following experiments and statistics analysis were blinded to groups’ allocations.

The selection of administration routes was based on the biological and physicochemical nature of each therapeutic agent. MSCs require intravenous (IV) delivery to ensure viability, systemic distribution, and homing to the injured kidney, as oral administration would result in their complete degradation within the gastrointestinal tract. Conversely, *Aloe vera* formulations (AVE, AVENPS, AVE-CSNPS) were administered orally to evaluate the potential for enhanced oral bioavailability- a key objective of the nano-encapsulation strategy. This route is the most feasible and patient-compliant for chronic phytotherapy. This design allowed for a direct assessment of whether nanotechnology could overcome the inherent oral bioavailability challenges of plant extracts and whether the resulting systemic effects could synergize with intravenously delivered MSCs.

Thirty five BALB/c mice were randomly allocated into seven groups (5 mice/group): group I: healthy negative control mice (NC), group II: untreated amikacin induced-kidney injured mice (positive control) (PC), group III: amikacin induced-kidney injured mice treated with MSCs (AMK-MSCs), group IV: amikacin induced-kidney injured mice treated with MSCs and AVE (AMK-MSCs/AVE), group V: amikacin induced-kidney injured mice treated with MSCs and AVENPS (AMK-MSCs/AVENPS), group VI: amikacin induced-kidney injured mice treated with MSCs and CSNPS (AMK-MSCs/CSNPS), and group VII: amikacin induced-kidney injured mice treated with MSCs and AVE-CSNPS (AMK-MSCs/AVE-CSNPS). Kidney injury induction was performed according to Batoo et al^58^., where amikacin (100 mg/kg/day) were intraperitoneally injected for ten successive days to mice (group II to VII). After confirmation of the induction of kidney injury, 2 × 10^6^ MSCs suspended in Dulbecco’s modified Eagles medium (DMEM) were injected intravenously into mice (group III to VII). According to the experimental groups AVE (250 mg/kg), AVENPS (250 mg/kg), CSNPS (250 mg/kg) and AVE-CSNPS (250 mg/kg) were orally administrated daily for four weeks to animals. For terminal anesthesia, animals were administered sodium pentobarbital (50 mg/kg) at the conclusion of the experiment. For preparation of serum samples, the blood was collected by cardiac puncture and centrifuged for 10 min at 1000 rpm to separate serum samples that were kept at −80 °C. For the preparation of kidney tissue homogenates, kidneys from all experimental groups were collected and homogenized with cold Tris-HCl solution (10 mmol, pH = 7.4) to prepare kidney tissue homogenates.

### Analysis of kidney function

In the serum samples, urea, creatinine and uric acid were measured by colorimetric kits (ab83362, ab65340 and ab65344, respectively; abcam, USA). For the collection of urine samples, the animals were placed separately in metabolic cages (with free access to water) overnight. Albumin was determined by colorimetric kit (abx298891, abbexa, UK) in the urine samples.

### Oxidative stress measurements

Oxidative stress was assessed by measuring malondialdehyde (MDA) and superoxide dismutase (SOD) levels in kidney tissue homogenates using ELISA kits (MBS741034 and MBS2707323, respectively; MyBioSource, USA). ELISA procedures followed the manufacturer’s guidelines and precautions.

### Immunological measurements

Inflammatory markers (IL-1β, TNF-α, CRP, and IL-6) and the anti-inflammatory marker (IL-4) were measured in kidney tissue homogenates using ELISA kits (ab197742, ab208348, ab222511, ab222503 and ab100710, respectively; Abcam, UK). ELISA procedures followed the manufacturer’s guidelines and precautions.

### Statistical analysis

Units were chosen based on the standard reporting conventions and sensitivity ranges for each specific assay kit to accurately reflect the biological concentrations of the respective biomarkers. The data were subjected to one-way analysis of variance (ANOVA) and given as means ± standard deviations (SD). The Bonferroni’s post hoc test was used to evaluate mean differences, and results were deemed statistically significant if the p value was less than 0.05. No animal was excluded from the analysis (*n* = 35).

## Results

### Phytochemical composition of AVE, AVENPS and AVE-CSNPS

#### GC-MS analysis

The GC-MS analysis showed significant differences in the phytochemical profiles among AVE, AVENPS, and AVE-CSNPS with respect to the abundance of key bioactive components. Hexadecanoic acid (palmitic acid) was the dominant compound in all samples (AVE: 14.73%, AVENPS: 13.60% and AVE-CSNPS: 14.53%). Also, methyl 12-hydroxyoctadecenoate showed a high abundance across samples (AVE: 14.3%, AVENPS: 13.4% and AVE-CSNPS: 14.22%). Most compounds showed < 15% variation between AVE and AVENPS. 10-Octadecenoic acid methyl ester maintained similar levels between AVE (11.51%) and AVENPS (11.07%). Stearic acid content was nearly identical in AVE and AVENPS (6.31% and 6.00%, respectively). In addition, slight decreases were observed in AVENPS for some compounds such as 6,9,12-octadecatrienoic acid methyl ester (AVE: 0.82% Vs. AVENPS: 0.51%), octadecanoic acid methyl ester (AVE: 1.49% Vs. AVENPS: 1.17%) and the permethylated glycolipid derivative (AVE: 0.16% Vs. AVENPS: 0.33%). AVE-CSNPS contained intermediate values for most phytochemical components between AVE and AVENPS (Table 1) with excellent recovery of key components like hexadecanoic acid (14.53%) and methyl 12-hydroxy-9(Z)-octadecenoate (14.22%). These findings demonstrated that the nanoencapsulation process effectively preserved the majority of phytochemical constituents from the original AVE, with the AVE-CSNPS showing minimal impact on overall compound composition. The maintained levels of bioactive fatty acids and other important phytochemicals suggested that the therapeutic potential of AVE was retained in both nanoparticle formulations.

#### HPLC analysis

HPLC quantification of phenolic compounds revealed excellent preservation of bioactive constituents in both AVENPS and AVE-CSNPS compared to the AVE (Table 2). The analysis demonstrated that the nanoencapsulation process maintained most phenolic compounds within 5% of their original concentrations, with ellagic acid showing the highest absolute concentration across all formulations (118.2, 117.31 and 118.06 µg/mL for AVE, AVENPS, and AVE-CSNPS). Catechin, another major component, was present at elevated levels (75.61, 74.29 and 75.09 µg/mL for AVE, AVENPS, and AVE-CSNPS), demonstrating the stability of flavan-3-ols during nanoparticles preparation. Chlorogenic acid, rutin, and kaempferol, representing three distinct phenolic classes, all showed excellent retention in the nanoparticles formulations. Chlorogenic acid maintained concentrations above 51 µg/mL in all samples (52.21 and 51.07 µg/mL in AVE and AVENPS, respectively), while rutin levels remained near 59 µg/mL (59.50 and 58.10 µg/mL in AVE and AVENPS, respectively). The flavonol kaempferol showed similar stability, with concentrations of 58.51 and 57.20 µg/mL in AVE and AVENPS, respectively. AVE-CSNPS showed intermediate values that closely matched the original extract, particularly for critical compounds like ferulic acid (46.41 and 46.20 µg/mL in AVE and AVE-CSNPS, respectively) and quercetin (38.37 and 38.20 µg/mL in AVE and AVE-CSNPS, respectively). Decreases in concentrations were observed for some compounds in AVENPS, including methyl gallate (14.41 µg/mL in AVE Vs. 13.61 µg/mL in AVENPS) and coumaric acid (18.55 µg/mL in AVE Vs. 17.26 µg/mL in AVENPS). However, these reductions were typically less than 10% and were partially recovered in AVE-CSNPS. The relative stability of more labile compounds like gallic acid (16.56 µg/mL in AVE Vs. 15.50 µg/mL in AVENPS) and caffeic acid (18.82 µg/mL in AVE Vs. 18.05 µg/mL in AVENPS) suggested that the nanoparticle preparation process provides substantial protection against phenolic degradation. These results collectively demonstrated that both nanoparticle formulations successfully preserve the diverse phenolic profile of Aloe vera, with the chitosan coating offering additional stabilization for certain compounds.

#### ICP-MS analysis

ICP-MS analysis revealed significant differences in the elemental composition of AVE, AVENPS and AVE-CSNPS (Table 3). Magnesium was the most abundant element in all samples, with concentrations of 8754.57, 8654.97, and 8728.83 ppm in AVE, AVENPS and AVE-CSNPS, demonstrating minimal loss during nanoparticle preparation. Similarly, potassium and calcium were present in high quantities, with AVE containing 4069.60 and 2579.97 ppm for potassium and calcium, respectively. While AVENPS showed slight reductions in these elements (4020.70 and 2352.20 ppm for potassium and calcium, respectively), AVE-CSNPS exhibited near-original levels (4052.23 and 2461.13 ppm for potassium and calcium, respectively), suggesting CSNPS helped in retaining the essential minerals. Trace elements such as zinc, iron, and manganese followed a similar trend, with AVENPS showing lower concentrations (85.46, 47.10, and 59.56 ppm for zinc, iron and manganese) compared to AVE (98.40, 52.33, and 68.23 ppm for zinc, iron and manganese). However, AVE-CSNPS partially restored these levels (93.76, 50.93, and 65.83 ppm for zinc, iron and manganese), indicating chitosan’s protective effect. Notably, copper experienced a more pronounced reduction in AVENPS (6.80 ppm) compared to AVE (9.56 ppm), but AVE-CSNPS nearly recovered the original concentration (9.23 ppm).

Heavy metal analysis showed that lead decreased from 1.30 ppm in AVE to 0.50 ppm in AVENPS, with AVE-CSNPS at an intermediate level (1.03 ppm). Nickel and cobalt were completely absent in AVENPS but present in trace amounts in AVE (1.73 and 0.66 ppm, respectively) and AVE-CSNPS (0 and 0.04 ppm, respectively). Chromium levels dropped significantly from 2.23 ppm in AVE to near-undetectable levels in both nanoparticle formulations. Silver and mercury were absent in all samples, confirming the safety of the formulations. These results demonstrated that while nanoparticle preparation reduced certain elemental concentrations, CSNPS loading mitigated these losses, particularly for essential minerals. The near-complete removal of toxic heavy metals (Ni, Co, Cr) in AVENPS and AVE-CSNPS suggested a purification effect during nanoencapsulation, while AVE-CSNPS maintained a balanced mineral profile closer to the original extract.

**Table 1:** Phytochemical compounds in AVE, AVENPS and AVE-CSNPS by GC-MS.

Compounds name	Formula	MW	Area%		
			AVE	AVENPS	AVE-CSNPS
**15**,**15’-Bi-1**,**4**,**7**,**10**,**13-pentaoxacyclohexadecane**	C_22_H_42_O_10_	466	0.16 ± 0.03	0.11 ± 0.02	0.14 ± 0.01
**Pentadecanoic acid**,** 14-methyl-**,** methyl ester**	C_17_H_34_O_2_	270	2.51 ± 0.10	2.36 ± 0.05	2.45 ± 0.13
**5**,**8**,**11-Heptadecatriynoic acid**,** methyl ester**	C_18_H_24_O_2_	272	0.30 ± 0.10	0.23 ± 0.05	0.28 ± 0.02
**Hexadecanoic acid**	C_16_H_32_O_2_	256	14.73 ± 0.20	13.60 ± 0.10	14.53 ± 0.25
**9**,**12-Octadecadienoic acid**,** methyl ester**	C_19_H_34_O_2_	294	3.40 ± 0.09	2.88 ± 0.09	3.40 ± 0.26
**10-Octadecenoic acid**,** methyl ester**	C_19_H_36_O_2_	296	11.51 ± 0.08	11.07 ± 0.13	11.42 ± 0.12
**Permethylated glycolipid derivative**	C_94_H_180_N_4_O_26_	1780	0.33 ± 0.15	0.16 ± 0.05	0.30 ± 0.10
**Octadecanoic acid**,** methyl ester**	C_19_H_38_O_2_	298	1.49 ± 0.11	1.17 ± 0.07	1.44 ± 0.05
**6**,**9**,**12-Octadecatrienoic acid**,** methyl ester**	C_19_H_32_O_2_	292	0.50 ± 0.08	0.32 ± 0.03	0.45 ± 0.05
**Androstan-17-one**,** 3-ethyl-3-hydroxy-**,** (5alpha)-**	C_21_H_34_O_2_	318	0.23 ± 0.07	0.16 ± 0.10	0.22 ± 0.07
**2-(7-Heptadecynyloxy)tetrahydro-2 H-pyran**	C_22_H_40_O_2_	336	0.24 ± 0.04	0.19 ± 0.01	0.21 ± 0.01
**9(Z)**,**12(Z)-Octadecadienoyl chloride**	C_18_H_31_ClO	298	2.17 ± 0.12	1.83 ± 0.07	2.13 ± 0.04
**Stearic acid**	C_18_H_36_O_2_	284	6.31 ± 0.07	6.00 ± 0.01	6.22 ± 0.26
**Octadecanoic acid**	C_18_H_36_O_2_	284	7.80 ± 0.09	7.61 ± 0.10	7.63 ± 0.05
**6**,**9**,**12-octadecatrienoic acid**,** methyl ester**	C_19_H_32_O_2_	292	0.82 ± 0.07	0.51 ± 0.02	0.66 ± 0.15
**Linolenic acid**,** 2-hydroxy-1-(hydroxymethyl)ethyl ester (Z**,** Z**,**Z)-**	C_21_H_36_O_4_	352	0.46 ± 0.04	0.39 ± 0.01	0.44 ± 0.05
**Methyl 12-Hydroxy-9(Z)-octadecenoate**	C_19_H_36_O_3_	312	14.30 ± 0.14	13.40 ± 0.49	14.22 ± 0.39
**Cyclopropanedecanoic acid**	C_22_H_40_O_4_	368	0.29 ± 0.01	0.17 ± 0.03	0.23 ± 0.06

Results were presented as mean ± SD.

**Table 2:** Phytochemical compounds in AVE, AVENPS and AVE-CSNPS by HPLC.

Compounds name	Formula	Conc. (µg/ml)		
		AVE	AVENPS	AVE-CSNPS
**Gallic acid**	C_7_H_6_O_5_	16.56 ± 0.05	15.50 ± 0.27	16.06 ± 0.12
**Chlorogenic acid**	C_16_H_18_O_9_	52.21 ± 0.10	51.07 ± 0.02	52.02 ± 0.06
**Catechin**	C_15_H_14_O_6_	75.61 ± 0.10	74.29 ± 0.44	75.09 ± 0.36
**Methyl gallate**	C_8_H_8_O_5_	14.41 ± 0.12	13.61 ± 0.10	14.13 ± 0.20
**Caffeic acid**	C_9_H_8_O_4_	18.82 ± 0.13	18.05 ± 0.06	18.49 ± 0.35
**Syringic acid**	C_9_H_10_O_5_	18.83 ± 0.06	17.61 ± 0.44	18.65 ± 0.13
**Pyrocatechol**	C_6_H_6_O_2_	39.27 ± 0.23	38.27 ± 0.30	39.03 ± 0.30
**Rutin**	C_27_H_30_O_16_	59.50 ± 0.43	58.10 ± 0.21	59.03 ± 0.06
**Ellagic acid**	C_14_H_6_O_8_	118.2 ± 0.27	117.31 ± 0.28	118.06 ± 0.20
**Coumaric acid**	C_9_H_8_O_3_	18.55 ± 0.13	17.26 ± 0.33	18.40 ± 0.20
**Vanillin**	C_8_H_8_O_3_	16.33 ± 0.48	16.13 ± 0.27	16.03 ± 0.25
**Ferulic acid**	C_10_H_10_O_4_	46.41 ± 0.25	45.70 ± 0.43	46.20 ± 0.60
**Naringenin**	C_15_H_12_O_5_	25.69 ± 0.17	24.49 ± 0.69	25.30 ± 0.65
**Daidzein**	C_15_H_10_O_4_	38.38 ± 0.16	37.63 ± 0.06	38.23 ± 0.15
**Quercetin**	C_15_H_10_O_7_	38.37 ± 0.15	37.22 ± 0.72	38.20 ± 0.20
**Cinnamic acid**	C_9_H_8_O_2_	8.966 ± 0.40	8.17 ± 0.32	8.53 ± 0.35
**Apigenin**	C_15_H_10_O_5_	41.50 ± 0.63	41.07 ± 0.31	41.06 ± 0.25
**Kaempferol**	C_15_H_10_O_6_	58.51 ± 0.46	57.20 ± 0.54	58.20 ± 0.30
**Hesperetin**	C_16_H_14_O_6_	24.72 ± 0.13	23.11 ± 0.21	24.50 ± 0.17

Results were presented as mean ± SD.

**Table 3:** Elemental analysis of AVE, AVENPS and AVE-CSNPS by ICP-MS.

Element	Concentration (ppm)		
	AVE	AVENPS	AVE-CSNPS
**Zinc (Zn)**	98.40 ± 0.80	85.46 ± 0.75	93.76 ± 1.38
**Calcium (Ca)**	2579.97 ± 0.40	2352.20 ± 2.01	2461.13 ± 7.22
**Iron (Fe)**	52.33 ± 1.33	47.10 ± 1.55	50.93 ± 0.85
**Magnesium (Mg)**	8754.57 ± 1.72	8654.97 ± 3.75	8728.83 ± 5.03
**Manganese (Mn)**	68.23 ± 1.22	59.56 ± 1.72	65.83 ± 0.95
**Copper (Cu)**	9.56 ± 0.61	6.80 ± 0.26	9.23 ± 0.35
**Phosphorus (P)**	852.40 ± 1.44	765.16 ± 2.64	828.96 ± 5.04
**Potassium (K)**	4069.60 ± 0.65	4020.70 ± 1.53	4052.23 ± 3.01
**Sodium (Na)**	35.20 ± 0.65	30.23 ± 0.90	32.56 ± 0.81
**Aluminum (Al)**	880.73 ± 1.66	798.93 ± 1.98	867.66 ± 2.17
**Lead (Pb)**	1.30 ± 0.40	0.50 ± 0.20	1.03 ± 0.15
**Silver (Ag)**	0	0	0
**Mercury (Hg)**	0	0	0
**Nickel (Ni)**	1.73 ± 0.56	0	0
**Cobalt (Co)**	0.66 ± 0.41	0	0.04 ± 0.05
**Chromium (Cr)**	2.23 ± 0.61	0.06 ± 0.11	0.03 ± 0.05

Results were presented as mean ± SD.

### Physical properties

#### Particle size distribution

**Fig. 1:**
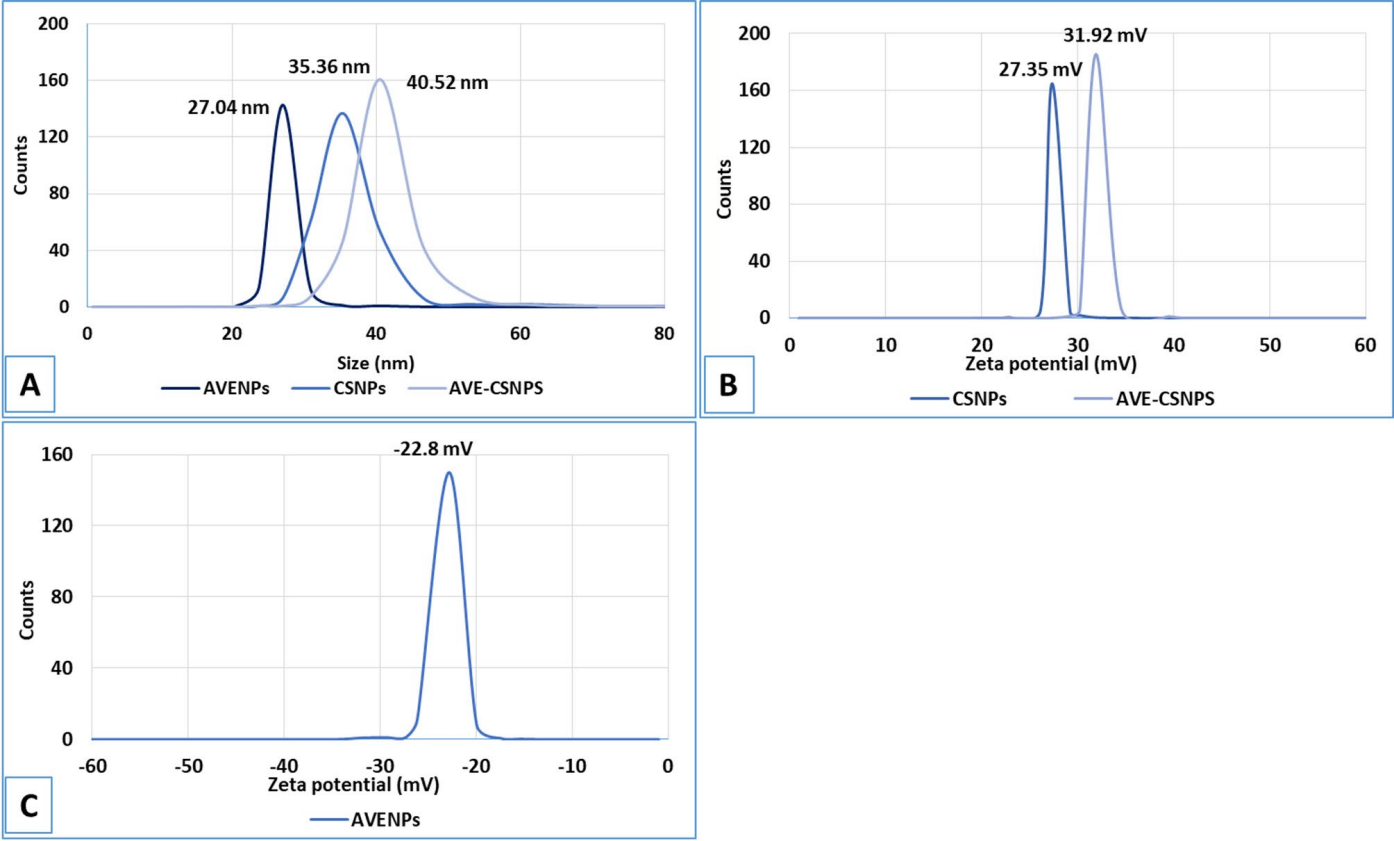
DLS (**A**) and zeta potential (**B** and **C**) of CSNPS, AVE-CSNPS and AVENPS.

DLS analysis showed distinct size distributions for the prepared nanoparticles (Fig. 1A). AVENPS exhibited an average hydrodynamic diameter of 27.04 nm, while unloaded CSNPS were slightly larger (35.36 nm). Upon loading AVE into the CSNPS, the particle size increased to 40.52 nm, confirming successful encapsulation. The narrow peaks in the size figure indicated a monodisperse distribution for all formulations, critical for reproducible biological interactions. The PDI values for AVENPS and AVE-CSNPS were below 0.3, indicating a narrow size distribution and good homogeneity.

#### Surface charge (zeta potential)

Zeta potential measurements in Figure (1 C) demonstrated that AVENPS had a moderately negative charge (− 22.8 mV), attributed to the anionic functional groups (e.g., carboxyl, hydroxyl) in AVE constituents. In contrast, CSNPS displayed a positive charge (27.35 mV) due to the protonated amino groups of chitosan. Remarkably, the AVE-CSNPS showed a higher positive zeta potential (31.92 mV) than unloaded CSNPS, suggesting that AVE components may have synergistically enhanced the surface charge, possibly through interactions that exposed additional cationic sites on chitosan or reduced aggregation-induced charge masking. This elevated positive charge further reinforced the formulation’s potential for mucoadhesion, colloidal stability, and cellular uptake.

#### EE%, DL% and retention efficiency

The phytochemical analysis revealed distinct patterns in bioactive compound recovery between nanoparticle formulations (Table 4). The AVE-CSNPS demonstrated superior EE%, with 81.4 ± 1.4% of TPC and 64.1 ± 2.1% of TFC successfully entrapped, corresponding to drug loading capacities of 14.5 ± 0.2% (TPC) and 11.4 ± 0.3% (TFC). In contrast, AVENPS, prepared via acid precipitation, showed 67.8 ± 1.3% TPC and 53.9 ± 1.8% TFC retention efficiency, indicating significant losses (~ 30–46%) of bioactive components during processing. Notably, chitosan coating enhanced phenolic compound stability (TPC in AVE-CSNPS: 37.5 ± 0.3 mg GAE/g vs. AVENPS: 31.2 ± 0.5 mg GAE/g), while flavonoids exhibited greater sensitivity to processing conditions (TFC in AVE-CSNPS: 26.2 ± 0.2 mg QE/g vs. AVENPS: 22.1 ± 1.2 mg QE/g). These results underscored chitosan’s protective role in nanoparticle formulations, mitigating bioactive degradation compared to carrier-free methods.

**Table 4:** Comparative analysis of TPC, TFC, EE%, DL%, and retention efficiency in AVE, AVENPS, and AVE-CSNPS.

Parameter	AVE	AVENPS	AVE-CSNPS
**TPC (mg GAE/g)**	46.0 ± 0.7	31.2 ± 0.5	37.5 ± 0.3
**TFC (mg QE/g)**	41.0 ± 0.9	22.1 ± 1.2	26.2 ± 0.2
**EE% (TPC)**	-	-	81.4 ± 1.4
**EE% (TFC)**	-	-	64.1 ± 2.1
**DL% (TPC)**	-	-	14.5 ± 0.2
**DL% (TFC)**	-	-	11.4 ± 0.3
**Retention efficiency (TPC)**	-	67.8 ± 1.3	-
**Retention efficiency (TFC)**	-	53.9 ± 1.8	-

Results were presented as mean ± SD.

### In vitro activities

Tables (S1-S5) that contained the *in vitro* activities were provided as supplementary materials.

#### Antioxidant activity (DPPH scavenging assay)

**Fig. 2:**
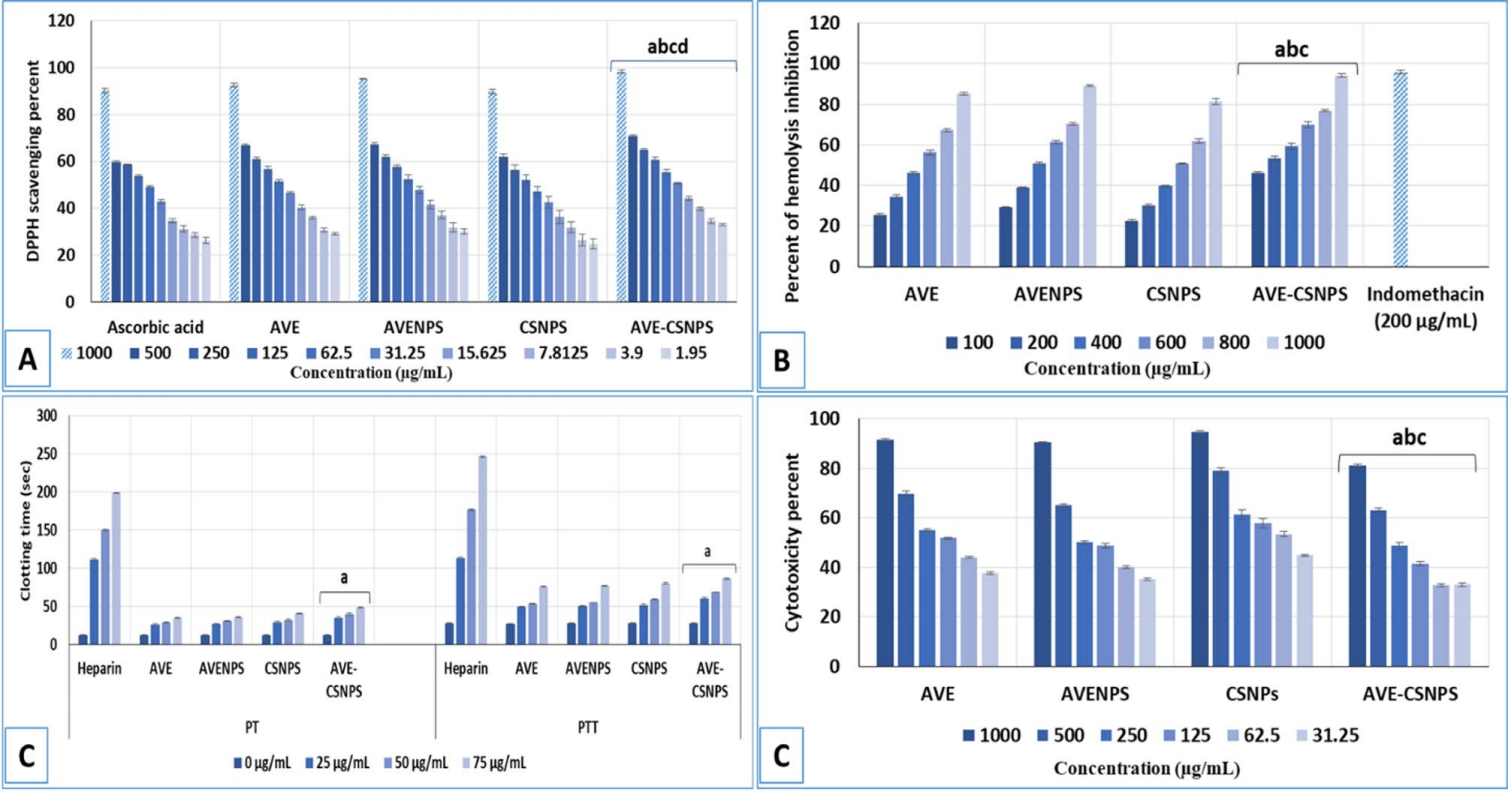
*In vitro* characterization of AVE, AVENPS, CSNPS and AVE-CSNPS showing the antioxidant activity **(A)** where a, b,c and d indicated significance (*P* < 0.05) in comparison to ascorbic acid (positive control), AVE, AVENPS and CSNPS, respectively; hemolysis inhibition **(B)** where a, b and c indicated significance (*P* < 0.05) in comparison to AVE, AVENPS and CSNPS, respectively; clotting time **(C)** where a indicated significance (*P* < 0.05) in comparison to heparin (positive control); the cytotoxicity activity **(D)** where a, b and c indicated significance (*P* < 0.05) in comparison to AVE, AVENPS and CSNPS, respectively.

 The antioxidant activity of AVE, AVENPS, CSNPS and AVE-CSNPS was determined using the DPPH assay (Fig. 2A). AVE-CSNPS showed the highest free radical scavenging activity at all tested concentrations significantly surpassing AVE, CSNPS and AVENPS. At high concentrations (1000 µg/mL), AVE-CSNPS showed near-maximal scavenging effect (98.2%), significantly higher than AVE (92.6%), AVENPS (95.1%) and ascorbic acid (90.1%). The negligible activity of unloaded CSNPS (89.9% relative to ascorbic acid) confirms that the enhanced antioxidant effect was specifically due to AVE’s bioactive components. At lower concentrations (≤ 250 µg/mL), AVE-CSNPS maintained significantly higher activity than AVENPS (e.g., 64.9% vs. 61.9% at 250 µg/mL) (Table S1). The gap widened further at 62.5 µg/mL (55.5% for AVE-CSNPS vs. 52.5% for AVENPS), highlighting chitosan’s role in enhancing AVE’s bioavailability. Notably, all tested samples showed dose-dependent behavior, but AVE-CSNPS achieved comparable efficacy to ascorbic acid (positive control) at lower concentrations, highlighting the synergistic effect of chitosan encapsulation on stabilizing AVE’s bioactive compounds.

#### Anti-inflammatory activity (hemolysis inhibition)

 Hemolysis inhibition assay (Fig. 2B) revealed that AVE-CSNPS induced the lowest hemolytic activity at all tested concentrations, with significant differences versus AVE, AVENPS, and CSNPS. AVE-CSNPS showed the strongest inhibition, reaching 94.2% at 1000 µg/mL, closely approaching the efficacy of indomethacin (95.8%) at a concentration of 200 µg/mL. In contrast, AVENPS and CSNPS showed lower effects (89.1% and 81.4% at 1000 µg/mL, respectively) (Table S2). At 200 µg/mL, AVE-CSNPS achieved 53.5% hemolysis inhibition, higher than AVENPS (38.9%) and CSNPS (30.2%). Even at lower concentrations (100 µg/mL), AVE-CSNPS (46.3%) surpassed AVENPS (29.1%) and CSNPS (22.7%), suggesting improved bioavailability due to chitosan encapsulation. Unloaded CSNPS showed moderate hemolysis, while AVE-CSNPS demonstrated superior biocompatibility, likely due to chitosan’s protective role in masking erythrocyte-membrane-disruptive components of AVE.

#### Anticoagulant activity (clotting time)

 AVE-CSNPS prolonged the clotting time (PT and PTT) more effectively than AVE or AVENPS at all tested concentrations (Fig. 2C), though heparin remained the most potent anticoagulant. While heparin (positive control) achieved maximal PT (198.7 s) and PTT (246.3 s) at 75 µg/mL, AVE-CSNPS reached ~ 24% (PT) and ~ 35% (PTT) of heparin’s activity at the same concentration (a notable effect for a natural composite). At 75 µg/mL, AVE-CSNPS extended PT to 48.5 s, significantly higher than AVENPS (35.6 s) and CS-NPS (40.5 s) (Table S3). For PTT, AVE-CSNPS exhibited the strongest effect among nanoparticles, reaching 86.6 s at 75 µg/mL vs. 77.1 s (AVENPS) and 80.3 s (CSNPS) (Table S4). This suggested chitosan’s cationic surface enhanced interactions with clotting factors, while AVE’s bioactive compounds further potentiate the anticoagulant activities.

#### Cytotoxicity evaluation

 Cytotoxicity MTT assay (Fig. 2D) indicated that AVE-CSNPS were the least toxic nanoparticles at all tested concentration, with significant differences compared to AVE, AVENPS, and CSNPS. At 1000 µg/mL, CSNPS exhibited the highest cytotoxicity (94.6%), significantly outperforming AVENPS (90.5%), AVE-CSNPS (81.0%), and AVE (91.7%). Across all tested concentrations (31.25–1000 µg/mL), AVE-CSNPS consistently maintained higher cell viability than AVENPS and CSNPS (Table S5). While AVE showed moderate cytotoxicity at lower doses (e.g., 51.8% at 125 µg/mL), AVE-CSNPS showed significantly lower cytotoxicity (41.4%) at the same concentration. This suggested that chitosan mitigated the potential toxic effects of raw AVE, likely by controlling the release of bioactive compounds.

### In vivo activities

#### Kidney function

**Fig. 3:**
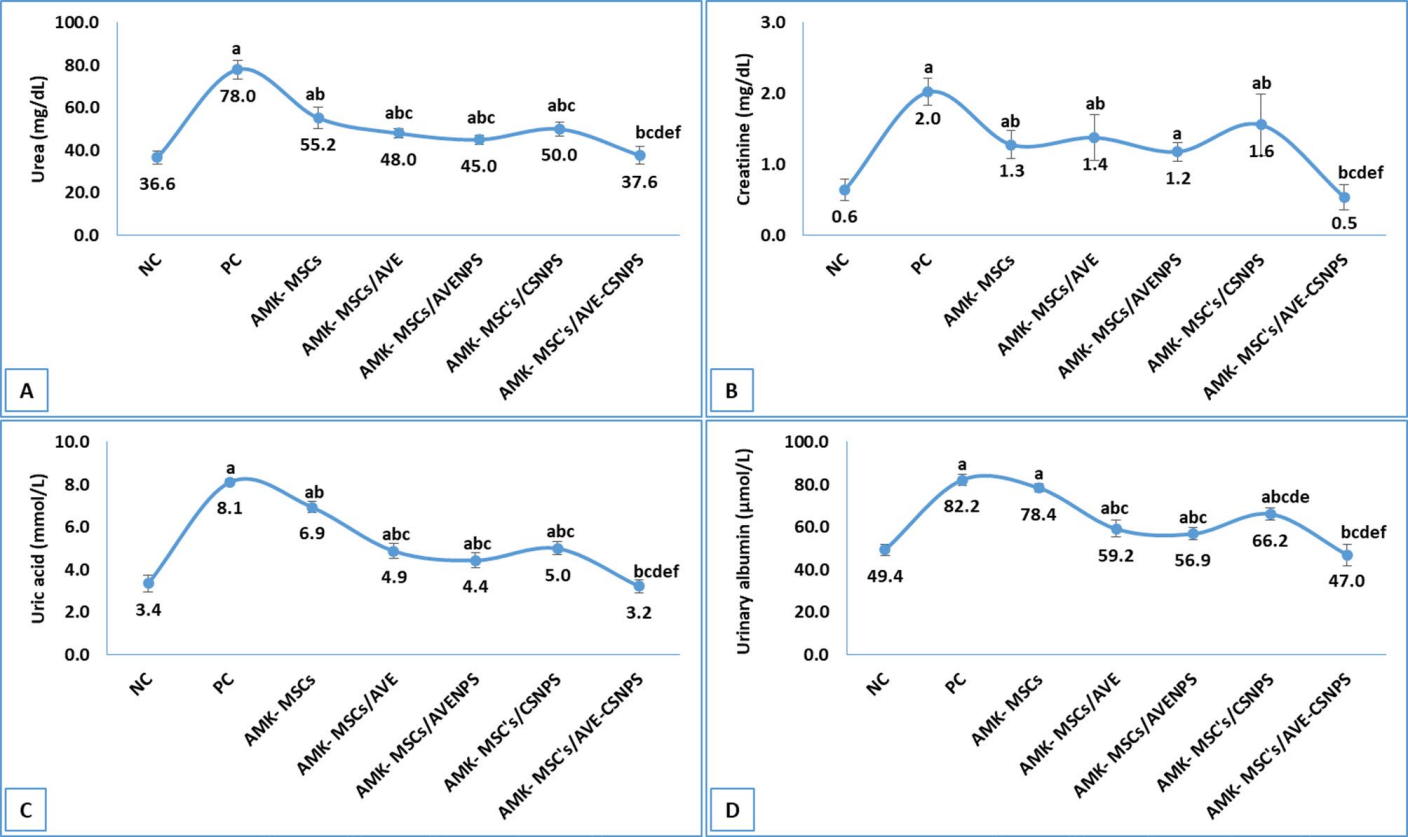
kidney function parameters showing levels of urea **(A)**, creatinine **(B)**, uric acid **(C)** and urinary albumin **(D)**. Data were represented as mean ± standard deviation (SD). a, b, c, d, e and f represents significance (*p* < 0.05) when compared to NC, PC, AMK-MSCs, AMK-MSCs/AVE, AMK-MSCs/AVENPS and AMK-MSCs/CSNPS, respectively.

The evaluation of kidney function parameters revealed significant improvements in all treatment groups compared to the PC group, with the AMK-MSCs/AVE-CSNPS group demonstrating the most pronounced therapeutic effects (Fig. 3). In the PC group, urea (78.0 ± 4.4 mg/dL), creatinine (2.0 ± 0.1 mg/dL), uric acid (8.1 ± 0.2 mmol/L), and urinary albumin (82.2 ± 2.5 µmol/L) levels were markedly elevated compared to the NC group, indicating severe kidney dysfunction. Treatment with MSCs alone (AMK-MSCs) partially ameliorated these abnormalities, reducing urea (55.2 ± 5.1 mg/dL), creatinine (1.3 ± 0.2 mg/dL), uric acid (6.9 ± 0.2 mmol/L), and urinary albumin (78.4 ± 2.0 µmol/L) levels (compared to PC). However, the combination therapies showed superior efficacy, particularly in AMK-MSCs/AVE-CSNPS group, which restored urea (37.6 ± 4.1 mg/dL), creatinine (0.5 ± 0.1 mg/dL), uric acid (3.2 ± 0.2 mmol/L), and urinary albumin (47.0 ± 5.1 µmol/L) to near-normal levels (compared to NC).

Notably, AMK-MSCs/AVENPS and AMK-MSCs/AVE also exhibited significant renal protection, with urea levels decreasing to 45.0 ± 2.2 mg/dL and 48.0 ± 2.2 mg/dL, respectively (compared to PC or AMK-MSCs). The AMK-MSCs/CSNPS group showed intermediate effects, suggesting that the combination of AVE-CSNPS with MSCs provided the most comprehensive renoprotection. These findings highlighted the synergistic potential of AVE-CSNPS in enhancing the therapeutic efficacy of MSCs for kidney injury, possibly through anti-inflammatory, antioxidant, and regenerative mechanisms.

#### Oxidative stress

**Fig. 4:**
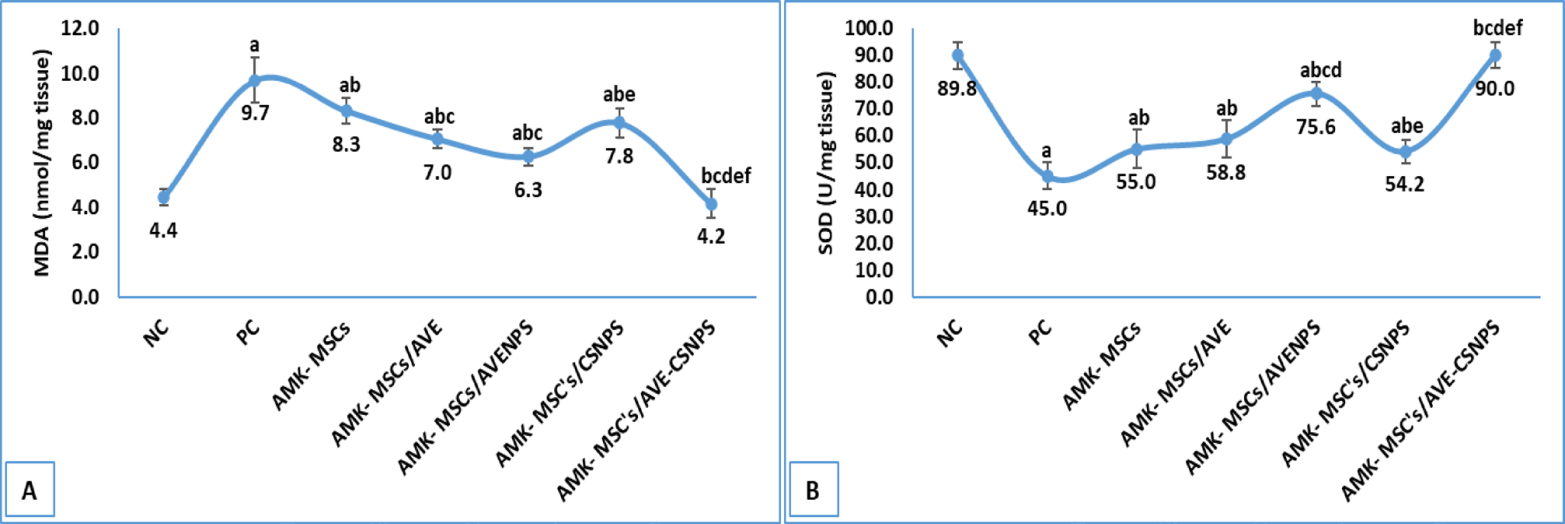
oxidative stress parameters in kidney tissue showing levels of MDA **(A)** and SOD **(B)**. Data were represented as mean ± standard deviation (SD). a, b, c, d, e and f represents significance (*p* < 0.05) when compared to NC, PC, AMK-MSCs, AMK-MSCs/AVE, AMK-MSCs/AVENPS and AMK-MSCs/CSNPS, respectively.

The assessment of oxidative stress parameters demonstrated significant therapeutic effects across all treatment groups, with the MSCs and AVE-CSNPS combination showing the most robust antioxidant activity (Fig. 4). In the PC group, MDA level was markedly elevated (9.7 ± 1.0 nmol/mg tissue), while SOD activity was significantly reduced (45.0 ± 7.2 U/mg tissue) compared to NC group, indicating severe oxidative damage. Treatment with MSCs alone partially mitigated these effects, lowering MDA to 8.3 ± 0.5 nmol/mg tissue and increasing SOD to 55.0 ± 7.2 U/mg tissue compared to positive control group. However, the combined therapies exhibited superior antioxidant effects, particularly AMK-MSCs/AVE-CSNPS group, which normalized both MDA (4.2 ± 0.6 nmol/mg tissue) and SOD (90.0 ± 4.9 U/mg tissue) to levels comparable to the normal control (NC).

The AMK-MSCs/AVENPS group also showed significant improvements, reducing MDA to 6.3 ± 0.4 nmol/mg tissue and restoring SOD to 75.6 ± 4.6 U/mg tissue (compared to PC or AMK-MSCs), suggesting that AVENPS enhance antioxidant defense mechanisms. In contrast, AMK-MSCs/CSNPS had more modest effects (MDA: 7.8 ± 0.6 nmol/mg tissue; SOD: 54.2 ± 4.3 U/mg tissue), underscoring the critical role of *Aloe vera* bioactives in combating oxidative stress. These findings highlight the synergistic potential of AVE-CSNPS in augmenting the antioxidant capacity of AMK-MSCs, likely through free radical scavenging and upregulation of endogenous antioxidant enzymes.

#### Inflammatory markers

**Fig. 5:**
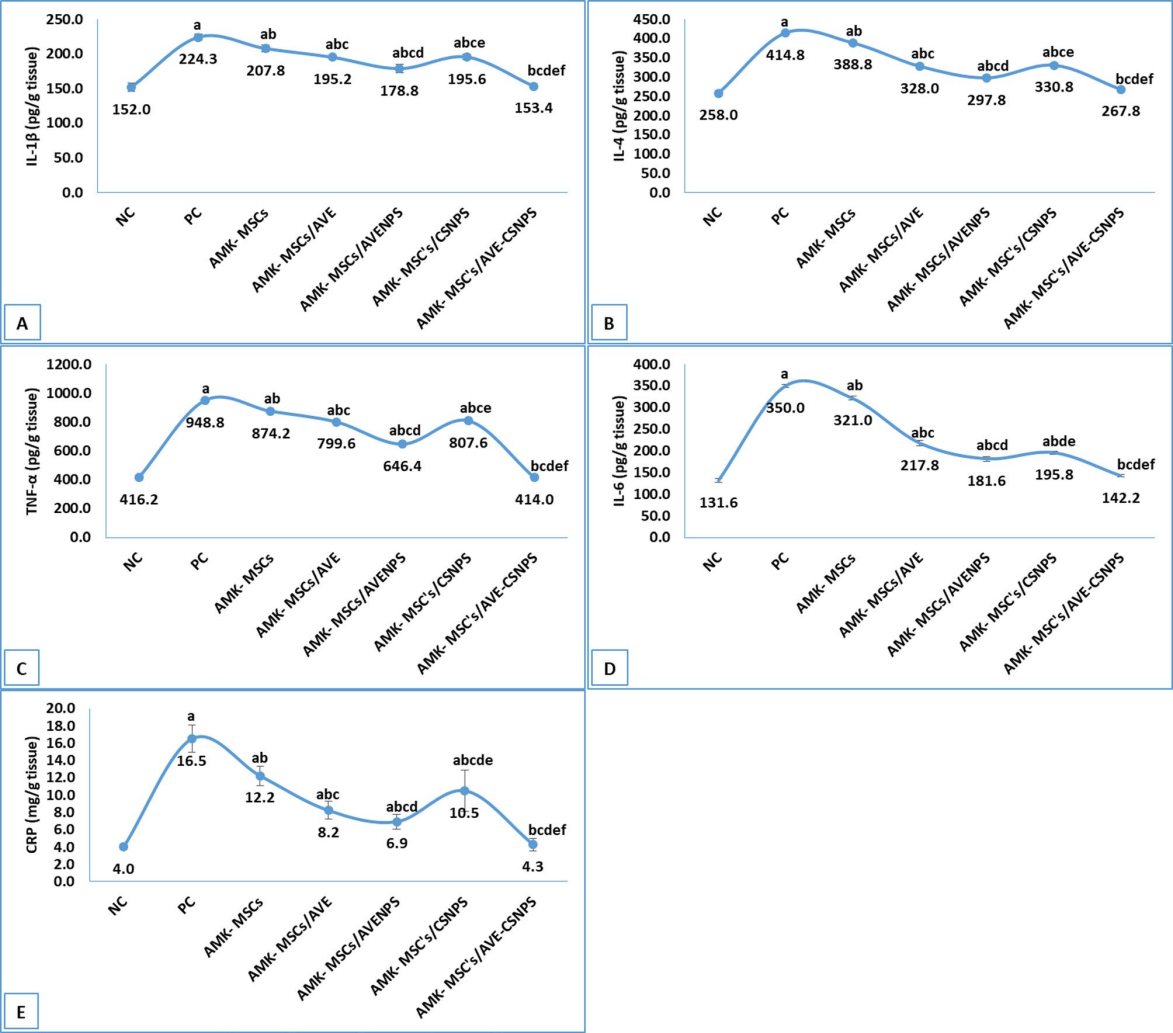
cytokine levels in kidney tissue showing IL-1β **(A)**, IL-4 **(B)**, TNF-α **(C)**, IL-6 **(D)** and CRP **(E)**. Data were represented as mean ± standard deviation (SD). a, b, c, d, e and f represents significance (*p* < 0.05) when compared to NC, PC, AMK-MSCs, AMK-MSCs/AVE, AMK-MSCs/AVENPS and AMK-MSCs/CSNPS, respectively.

The evaluation of inflammatory mediators revealed a robust anti-inflammatory effect of the combined MSCs and AVE-CSNPS therapy, demonstrating superior modulation of both pro- and anti-inflammatory cytokines compared to other treatment groups (Fig. 5). The PC group exhibited significantly elevated levels of pro-inflammatory markers, including IL-1β (224.3 ± 4.1 pg/g tissue), TNF-α (948.8 ± 3.9 pg/g tissue), IL-6 (350.0 ± 3.3 pg/g tissue), and CRP (16.5 ± 1.5 mg/g tissue), along with increased anti-inflammatory IL-4 (414.8 ± 4.6 pg/g tissue) compared to NC group, indicating a severe inflammatory response. While MSCs monotherapy partially attenuated this response (e.g., TNF-α: 874.2 ± 4.6 pg/g tissue; IL-6: 321.0 ± 4.5 pg/g tissue; IL-1β: 207.8 ± 5.0 pg/g tissue; CRP: 12.2 ± 1.1 mg/g tissue), the combination therapies showed substantially greater efficacy. Remarkably, combined MSCs and AVE-CSNPS normalized all inflammatory parameters to near-baseline levels (IL-1β: 153.4 ± 3.6 pg/g tissue; TNF-α: 414.0 ± 7.8 pg/g tissue; IL-6: 142.2 ± 3.1 pg/g tissue; CRP: 4.3 ± 0.7 mg/g tissue), with IL-4 (267.8 ± 3.1 pg/g tissue) also returning to normal physiological ranges.

The AMK-MSCs/AVENPS group showed particularly strong suppression of pro-inflammatory cytokines (TNF-α: 646.4 ± 6.8 pg/g tissue; IL-6: 181.6 ± 5.4 pg/g tissue), suggesting enhanced bioavailability of *Aloe vera*’s anti-inflammatory compounds in nanoparticle form. In contrast, AMK-MSCs/CSNPS exhibited more moderate effects (TNF-α: 807.6 ± 4.2 pg/g tissue; IL-6: 195.8 ± 3.7 pg/g tissue), underscoring the critical role of *Aloe vera* bioactives in inflammation control. The differential regulation of IL-4 across groups suggested that AVE-CSNPS may promote a balanced immune response rather than generalized immunosuppression.

## Discussion

Traditional herbal medicine has utilized plant-derived phytochemicals for centuries due to their therapeutic benefits, minimal side effects, and cost-effectiveness^59^. These bioactive compounds demonstrate significant pharmacological potential against microbial infections and chronic diseases like cancer and diabetes^60^. However, their clinical application faces substantial challenges, including poor bioavailability, low absorption rates, lack of target specificity, instability in acidic environments, and the need for high therapeutic doses^61^. Nanotechnology offers innovative solutions to overcome these limitations by enhancing phytochemical delivery systems. Nanoparticle formulations can significantly improve plant-derived compounds’ solubility, stability, and absorption while enabling controlled release kinetics^62^. The unique properties of nanocarriers allow for both hydrophilic and hydrophobic drug loading, multiple administration routes, and extended shelf life - collectively enhancing therapeutic efficacy while reducing required dosages^63^. Particularly for oral delivery, nanoencapsulation protects phytochemicals from gastric degradation while facilitating intestinal absorption, as demonstrated in numerous studies of herbal medicine nanoformulations^64^.

The present study successfully developed and characterized two nanoformulations of *Aloe vera* leaves, AVENPS and AVE-CSNPS, with both formulations demonstrating excellent preservation of the extract’s bioactive constituents. The comprehensive phytochemical characterization of AVE, AVENPS, and AVE-CSNPS revealed essential insights into the impact of nanoencapsulation on bioactive compound preservation. GC-MS analysis demonstrated remarkable retention of key fatty acids like hexadecanoic acid (palmitic acid) and methyl 12-hydroxyoctadecenoate across all formulations, with less than 15% variation from the original extract, consistent with previous findings that nanoparticle encapsulation can effectively preserve plant-derived lipids^65^. The HPLC results further confirmed this stability, showing excellent preservation (>95%) of phenolic compounds, including ellagic acid, catechin, and chlorogenic acid, aligning with reports that nanoencapsulation protects labile phytochemicals from degradation^66^. Notably, the chitosan-coated nanoparticles (AVE-CSNPS) showed superior recovery of several compounds compared to carrier-free AVENPS, particularly for sensitive phenolics like gallic and caffeic acids, supporting chitosan’s established role in stabilizing plant metabolites through electrostatic interactions and physical barrier formation^67^. The ICP-MS data revealed an interesting dual effect of nanoencapsulation; while some essential minerals (Mg, K, Ca) were well-preserved in both nanoparticle types, the process significantly reduced toxic heavy metal content (Pb, Ni, Co, Cr), suggesting an inherent purification effect that enhanced safety without compromising therapeutic mineral content, a phenomenon previously observed in plant extract nanoformulations^68^. The superior mineral retention in AVE-CSNPS (particularly for Zn, Fe, and Cu) compared to AVENPS underscored chitosan’s chelating properties and its ability to maintain the elemental profile closer to the native extract^69^. These collective findings demonstrated that while both nanoparticle formulations successfully preserved the major phytochemical constituents of AVE, the chitosan-based system offered additional advantages in terms of compound stabilization and mineral retention, likely contributing to its enhanced biological activities observed in subsequent experiments. The maintained phytochemical profile and reduced heavy metal content positioned these nanoformulations, particularly AVE-CSNPS, as promising candidates for therapeutic applications where both efficacy and safety were paramount.

The nanoparticle yield from the AVENPS process was sufficient for the comprehensive preclinical assessments conducted in this study. However, it is acknowledged that the acid precipitation method, while ideal for generating a carrier-free comparator for mechanistic studies, presents challenges for large-scale therapeutic production. These include the need for extensive purification (dialysis) and its relatively lower retention efficiency. In contrast, the ionic gelation method employed for AVE-CSNPS is recognized for its greater potential for scalability and was a key factor in its selection as the lead therapeutic candidate from this work.

The physicochemical characterization of the nanoparticles revealed crucial structure-function relationships that determined their therapeutic potential. Dynamic light scattering analysis showed that AVE-CSNPS (40.52 nm) were larger than both AVENPS (27.04 nm) and unloaded CSNPS (35.36 nm), consistent with successful encapsulation of AVE within the chitosan matrix as reported by Abreu et al^70^. in their work on plant extract-loaded nanoparticles. The monodisperse size distribution (PDI < 0.3) observed in all formulations was particularly advantageous for biological applications, as narrow size distributions enhance reproducibility and predictable biodistribution^71^. The zeta potential results demonstrated a remarkable increase in positive charge from + 27.35 mV (unloaded CSNPS) to + 31.92 mV (AVE-CSNPS), suggesting electrostatic interactions between AVE’s anionic groups and chitosan’s protonated amines that may create a more stable colloidal system, as previously observed by Szymańska et al^72^. with clotrimazole-loaded chitosan nanoparticles. This enhanced surface charge likely contributed to the improved mucoadhesion and cellular uptake reported for similarly charged nanoparticles^73^. The encapsulation efficiency data revealed chitosan’s superior protective capacity, with AVE-CSNPS achieving significantly higher EE% for both total phenolics (81.4%) and flavonoids (64.1%) compared to AVENPS, corroborating findings by Detsi et al^74^. that chitosan encapsulation better-preserved plant bioactive compounds during processing. The differential stability observed between phenolic compounds and flavonoids aligned with previous reports that flavonoid glycosides were particularly susceptible to degradation during nanoparticle preparation^75^, highlighting the importance of protective matrices like chitosan for maintaining phytochemical integrity. The high encapsulation efficiency (EE% >81% for TPC and >64% for TFC) demonstrated the successful entrapment of *Aloe vera* bioactives within the chitosan matrix, attributable to strong electrostatic interactions. The resultant drug loading (DL% ~14.5% for TPC and ~ 11.4% for TFC) indicated a favorable bioactive-to-carrier ratio, ensuring a therapeutically significant payload was delivered. These optimal EE% and DL% values were critical prerequisites for the enhanced bioavailability and efficacy observed in the subsequent *in vivo* studies.

The *in vitro* biological evaluations demonstrated the superior performance of AVE-CSNPS across multiple therapeutic parameters, validating the advantages of chitosan encapsulation. The remarkable antioxidant capacity of AVE-CSNPS (98.2% DPPH scavenging at 1000 µg/mL) significantly exceeded both free AVE and AVENPS, consistent with findings by Ashraf et al.^47^, who reported enhanced antioxidant activity of chitosan-encapsulated plant phenolics due to improved stability and controlled release. The anti-inflammatory assessment revealed AVE-CSNPS’ exceptional hemolysis inhibition (94.2%), approaching the standard indomethacin, which aligned with the work of Tarek et al^53^. demonstrating chitosan’s ability to potentiate anti-inflammatory effects through membrane stabilization and reduced oxidative stress. The anticoagulant results showed AVE-CSNPS achieved 24–35% of heparin’s activity, noteworthy for a natural formulation, supporting previous observations by Gheorghiță et al^76^. regarding chitosan’s intrinsic anticoagulant properties through factor XII activation. Cytotoxicity assessments revealed AVE-CSNPS’ superior biocompatibility (81% cell viability at 1000 µg/mL), corroborating studies by Liu et al.^77^, Ashraf et al^47^. and Tarek et al^53^. that chitosan encapsulation reduced phytochemical toxicity through controlled release kinetics. These collective findings established AVE-CSNPS as a multifunctional therapeutic platform combining enhanced bioactivity with improved safety profiles.

The therapeutic potential of MSCs is significantly constrained by several critical limitations, including poor post-transplantation survival rates, inefficient homing to injury sites following systemic administration, variable secretory activity dependent on local cytokine environments, and incompletely understood mechanisms of action^78–80^. These challenges are compounded by the complex interactions between NPs and stem cells, which can yield either beneficial or detrimental outcomes depending on NP characteristics. While some studies demonstrated NP-enhanced MSCs functionality, others revealed significant toxicity concerns - particularly with metallic NPs. For instance, silver nanoparticles (Ag NPs) were shown to induce transcriptomic alterations in mouse embryonic stem cells (ESCs) through reactive oxygen species (ROS)-mediated pathways, potentially compromising their regenerative capacity^81,82^. Similarly, gold nanoparticles (Au NPs) exhibited size- and surface chemistry-dependent toxicity in human ESCs^83^, though paradoxically enhanced dopaminergic differentiation in mouse ESCs in other studies^84^. This dichotomy underscores the critical importance of careful NPs selection and design when combining with stem cell therapies. Notably, plant-derived NPs and biocompatible carriers like chitosan demonstrate more favorable profiles, as evidenced by improved MSCs viability and synergistic therapeutic effects in animal models^85–87^. The optimal integration of MSCs with phytochemical-loaded NPs must therefore balance enhanced bioactivity against potential cytotoxicity, requiring meticulous optimization of NPs composition, size, and surface properties to maximize therapeutic outcomes while minimizing adverse effects on stem cell function.

The *in vivo* results demonstrated remarkable synergistic effects between MSCs and AVE-CSNPS in ameliorating amikacin-induced kidney injury, with the combination therapy showing superior renoprotective effects compared to monotherapies. Notably, the observed bioactivity of AVE-CSNPS is attributed to a synergistic combination of the inherent therapeutic properties of chitosan and the enhanced delivery of *Aloe vera* bioactives. The intrinsic antioxidant and anti-inflammatory activities of the chitosan carrier itself, as evidenced by the effects in the CSNPS control group, contribute to the overall efficacy, while the encapsulation process significantly improves the bioavailability and stability of the *Aloe vera *extract. The hydrodynamic size and low polydispersity index (PDI < 0.3) of AVE-CSNPS were critical quality attributes reflecting a superior formulation. The nanoscale size (~ 40 nm) is optimal for cellular uptake and bioavailability, while the monodisperse size distribution (indicated by the low PDI) signifies a homogeneous nanoparticle population, ensuring batch-to-batch reproducibility and predictable *in vivo* behavior essential for therapeutic efficacy. The AMK-MSCs/AVE-CSNPS group exhibited the most significant improvement in kidney function markers, nearly normalizing urea (37.6 ± 4.1 mg/dL), creatinine (0.5 ± 0.1 mg/dL), and other parameters, consistent with findings by Wang et al^88^. who reported enhanced therapeutic effects when combining MSCs with nanoparticle-encapsulated natural compounds. The oxidative stress results revealed that AVE-CSNPS significantly boosted the antioxidant capacity of MSCs, reducing MDA levels by 56.7% and increasing SOD activity by 100% compared to the positive control, supporting previous observations by Herdiana et al^89^. regarding chitosan nanoparticles’ ability to enhance cellular antioxidant defenses. The anti-inflammatory effects were particularly striking, with AVE-CSNPS plus MSCs reducing pro-inflammatory cytokines (TNF-α, IL-6) to near-normal levels while maintaining physiological IL-4 concentrations, mirroring results from Tang et al^90^. demonstrating chitosan’s immunomodulatory properties in kidney injury models. The superior performance of AVE-CSNPS over both AVENPS and blank CSNPS highlights the dual advantage of chitosan’s intrinsic bioactivity and its ability to protect and deliver *Aloe vera*’s therapeutic compounds, as previously observed in acute lung injury models by El-Emam et al.^54^. These findings collectively suggested that AVE-CSNPS enhance MSCs survival and function and create a regenerative microenvironment through combined antioxidant, anti-inflammatory, and possibly direct tissue-repair mechanisms. The carrier-free AVENPS formulation was deemed less favorable for bioactive delivery compared to AVE-CSNPS due to its significantly lower retention efficiency, which resulted in substantial loss of phytochemicals during processing. This was compounded by its inferior physicochemical stability, evidenced by a lower zeta potential, and its consistently reduced performance in both in vitro bioactivity assays and in vivo therapeutic efficacy.

Notably, the superior *in vivo* efficacy of both nanoformulations, particularly AVE-CSNPS, was achieved despite a lower nominal dose of bioactive components compared to the crude extract AVE, as quantified by TPC/TFC retention. This demonstrates that the enhanced therapeutic outcome is not a function of bioactive payload alone, but rather a result of significantly improved bioavailability, stability, and targeted delivery conferred by the nano-encapsulation process itself. Collectively, the chitosan-based encapsulation (AVE-CSNPS) provided distinct advantages over the carrier-free nanoparticles (AVENPS). The chitosan carrier functions as an active enabling technology that drastically amplifies the therapeutic utility of the encapsulated compounds. These included superior protection of labile phytochemicals, enhanced colloidal stability due to a high positive zeta potential, and significantly improved *in vitro* bioactivity across antioxidant, anti-inflammatory, and anticoagulant assays. Most importantly, these superior physicochemical properties translated into enhanced therapeutic efficacy *in vivo*, as evidenced by the significantly better recovery of renal function and reduction in oxidative stress and inflammation in the AVE-CSNPS treated group, underscoring the critical role of the chitosan carrier in boosting bioavailability and synergistic action.

In conclusion, the study demonstrated that AVE-CSNPS synergistically enhanced MSCs therapy for kidney injury by preserving the phytochemical integrity, improving bioavailability, and amplifying antioxidant/anti-inflammatory effects more than AVE or AVENPS. While this study demonstrated the synergistic effect of AVE-CSNPS and MSCs therapy for kidney injury, certain strategic considerations need further exploration. The current findings were based on a well-established amikacin-induced AKI model, which, while highly reproducible, does not fully recapitulate the complexity of chronic kidney disease progression in humans. Additionally, the 4-week treatment period, though sufficient to observe significant therapeutic effects, may not reveal long-term outcomes such as tissue remodeling or potential late-stage immune responses. Finally, the doses were administered based on the equivalent mass of the initial crude extract; future studies normalizing doses to bioactive content could provide further mechanistic insight. These aspects, however, did not diminish the validity of the observed synergistic mechanisms but rather highlighted opportunities for future research. Subsequent studies could extend these findings by employing progressive fibrosis models to evaluate long-term renoprotection and investigating dose-escalation protocols to optimize the therapeutic period with high-resolution histopathological analysis. Such targeted investigations would further strengthen the translational potential of this combinatorial approach while building upon the robust foundation established in this work.

## Supplementary Information

Below is the link to the electronic supplementary material.

Supplementary Material 1

## Data Availability

The datasets used and/or analyzed during the current study are available from the corresponding author on reasonable request.

## Abbreviations

DPPH: 1, 1-diphenyl-2-picryl hydrazyl
MTT: 3-(4,5-dimethylthiazol-2-yl)-2-5-diphenyltetrazolium bromide
AKI: Acute kidney injury
AVE: *Aloe vera* extract
AVE-CSNPS: *Aloe vera* extract loaded chitosan nanoparticles
AVENPS: *Aloe vera* extract nanoparticles
AMK: Amikacin
AGs: Aminoglycosides
ARBs: Angiotensin receptor blockers
ACEIs: Angiotensin-converting enzyme inhibitors
CRP: C-reactive protein
DMSO: Dimethyl sulfoxide
DL: Drug loading
DMEM: Dulbecco’s modified Eagles medium
DLS: Dynamic light scattering
EE: Encapsulation efficiency
GC-MS: Gas chromatography-mass spectroscopy
ICP: Inductively coupled plasma
CU-IACUC: Institutional animal care and use committee of Cairo University
IL: Interleukin
MDA: Malondialdehyde
MSCs: Mesenchymal stem cells
NPs: Nanoparticles
NODCAR: National Organization for Drug Control and Research
NSAIDs: Nonsteroidal anti-inflammatory drugs
ANOVA: One way analysis of variance
PTT: Partial thromboplastin time
PCGII: Positive control group II
PT: Prothrombin time
TPP: Sodium tripolyphosphate
SOD: Superoxide dismutase
TFC: Total flavonoid content
TPC: Total phenolic content
TNF- a: Tumor necrosis factor
