# Supplementary material for: Chitosan-encapsulated Aloe vera nanoparticles outperform carrier-free forms in enhancing MSCs therapy for amikacin nephrotoxicity
Source: Sci Rep. 2025 Oct 1;15:34292. doi: 10.1038/s41598-025-20918-6 (PMC12489135; doi:10.1038/s41598-025-20918-6)
Supplement: Supplementary file 1 — Supplementary Material 1 [file 41598_2025_20918_MOESM1_ESM.docx]

***In vitro* results:**

Table S1: The antioxidant activity (DPPH scavenging effect).

| **Sample**  **Conc.**  **(µg/ml)** | **Ascorbic acid** | **AVE** | **AVENPS** | **CSNPS** | **AVE-CSNPS** |
| --- | --- | --- | --- | --- | --- |
| **1000** | 90.06±0.85 | 92.63±0.85 | 95.16±0.15 | 89.86±0.98 | 98.23±0.58^abcd^ |
| **500** | 59.73±0.55 | 66.93±0.41 | 67.33±0.65 | 62.03±1.05 | 70.90±0.36^abcd^ |
| **250** | 58.56±0.40 | 60.96±0.80 | 61.86±1.09 | 56.56±1.95 | 64.93±0.65^abcd^ |
| **125** | 53.73±0.49 | 56.76±1.13 | 57.56±1.06 | 52.26±1.93 | 60.73±0.96^abcd^ |
| **62.5** | 49.33±0.25 | 51.53±0.81 | 52.53±1.62 | 47.23±2.05 | 55.50±1.01^abcd^ |
| **31.25** | 42.96±0.81 | 46.70±0.34 | 47.93±1.55 | 42.63±2.34 | 50.66±0.35^abcd^ |
| **15.625** | 34.66±0.87 | 40.36±0.97 | 41.66±1.85 | 36.36±2.71 | 44.33±0.80^abcd^ |
| **7.8125** | 31.16±1.28 | 36.00±0.45 | 37.20±1.58 | 31.90±2.25 | 39.96±0.65^abcd^ |
| **3.9** | 28.73±0.90 | 30.70±0.86 | 31.83±1.96 | 26.53±2.61 | 34.66±1.02^abcd^ |
| **1.95** | 26.33±1.36 | 29.20±0.43 | 30.16±1.26 | 24.86±2.01 | 33.16±0.45^abcd^ |

Data were represented as mean±standard deviation (SD). a,b,c and d indicated significance (P<0.05) in comparison to ascorbic acid (positive control), AVE, AVENPS and CSNPS, respectively.

Table S2: The anti-inflammatory activity (hemolysis inhibition assay).

| **Sample**  **Conc.**  **(µg/ml)** | **AVE** | **AVENPS** | **CSNPS** | **AVE-CSNPS** |
| --- | --- | --- | --- | --- |
| **100** | 25.4±0.9 | 29.13±0.32 | 22.70±0.45 | 46.30±0.36^abc^ |
| **200** | 34.56±0.70 | 38.96±0.45 | 30.20±0.75 | 53.56±0.97^abc^ |
| **400** | 46.20±0.79 | 51.06±0.61 | 39.80±0.45 | 59.46±1.55^abc^ |
| **600** | 56.16±1.41 | 61.33±0.95 | 51.06±0.20 | 69.96±1.55^abc^ |
| **800** | 67.16±0.70 | 70.40±0.72 | 61.90±1.08 | 76.80±0.43^abc^ |
| **1000** | 85.23±0.63 | 89.06±0.40 | 81.36±1.59 | 94.20±0.85^abc^ |

Data were represented as mean±standard deviation (SD). a,b and c indicated significance (*P*<0.05) in comparison to AVE, AVENPS and CSNPS, respectively.

Table S3: The anticoagulant activity (PT assay).

| **Sample**  **Conc.**  **(µg/ml)** | **PT** | | | | |
| --- | --- | --- | --- | --- | --- |
|  | **Heparin** | **AVE** | **AVENPS** | **CSNPS** | **AVE-CSNPS** |
| **0 µg/ml** | 12.26±0.35 | 12.30±0.40 | 12.06±0.45 | 12.16±0.61 | 12.36±0.30^a^ |
| **25 µg/ml** | 111.23±1.16 | 26.3±1.11 | 27.16±0.15 | 29.30±0.91 | 35.50±0.70^a^ |
| **50 µg/ml** | 150.26±0.73 | 28.9±0.50 | 30.43±0.49 | 32.33±0.76 | 40.06±1.45^a^ |
| **75 µg/ml** | 198.70±0.75 | 35.03±0.50 | 35.60±0.72 | 40.50±0.75 | 48.46±0.65^a^ |

Data were represented as mean±standard deviation (SD). a,b,c and d indicated significance (P<0.05) in comparison to heparin (positive control), AVE, AVENPS and CSNPS, respectively.

Table S4: The anticoagulant activity (PTT assay).

| **Sample**  **Conc.**  **(µg/ml)** | **PTT** | | | | |
| --- | --- | --- | --- | --- | --- |
|  | **Heparin** | **AVE** | **AVENPS** | **CSNPS** | **AVE-CSNPS** |
| **0 µg/ml** | 27.83±0.55 | 27.30±0.55 | 27.96±0.64 | 28.13±0.66 | 27.63±0.86^a^ |
| **25 µg/ml** | 113.70±0.62 | 49.23±0.35 | 50.66±0.30 | 51.90±0.70 | 60.66±1.30^a^ |
| **50 µg/ml** | 176.86±1.02 | 53.80±0.45 | 55.06±0.30 | 59.60±0.62 | 68.70±0.30^a^ |
| **75 µg/ml** | 246.30±1.17 | 75.90±0.62 | 77.13±0.70 | 80.30±1.21 | 86.56±0.81^a^ |

Data were represented as mean±standard deviation (SD). a,b,c and d indicated significance (P<0.05) in comparison to heparin (positive control), AVE, AVENPS and CSNPS, respectively.

Table S5: the cytotoxicity activity (MTT assay).

| **Sample**  **Conc.**  **(µg/ml)** | **AVE** | **AVENPS** | **CSNPS** | **AVE-CSNPS** |
| --- | --- | --- | --- | --- |
| **1000** | 91.70±0.45 | 90.53±0.32 | 94.56±0.64 | 81.00±0.62 ^abc^ |
| **500** | 69.73±1.30 | 65.03±0.75 | 79.13±1.19 | 63.06±0.70 ^abc^ |
| **250** | 55.10±0.62 | 50.10±0.70 | 61.40±1.90 | 48.70±1.34 ^abc^ |
| **125** | 51.76±0.45 | 48.66±0.90 | 57.73±1.96 | 41.43±0.80 ^abc^ |
| **62.5** | 43.96±0.30 | 40.06±0.58 | 53.73±1.11 | 32.86±0.57 ^abc^ |
| **31.25** | 37.70±0.43 | 35.10±0.55 | 44.83±0.35 | 33.03±0.77 ^abc^ |

Data were represented as mean±standard deviation (SD). a,b and c indicated significance (*P*<0.05) in comparison to AVE, AVENPS and CSNPS, respectively.
